# Dual-energy CT collagen density mapping of wrist ligaments reveals tissue remodeling in CPPD patients: first results from a clinical cohort

**DOI:** 10.1007/s00256-020-03580-z

**Published:** 2020-08-15

**Authors:** Katharina Ziegeler, Sophia-Theresa Richter, Sandra Hermann, Kay Geert A. Hermann, Bernd Hamm, Torsten Diekhoff

**Affiliations:** 1 Department of Radiology, Charité–Universitätsmedizin, Charitéplatz 1, 10117 Berlin, Germany; 2 Department of Rheumatology and Clinical Immunology, Charité–Universitätsmedizin, Charitéplatz 1, Berlin, 10117 Germany

**Keywords:** Chondrocalcinosis, Ligaments, Wrist, Dual-energy computed tomography, Collagen

## Abstract

**Objectives:**

To evaluate differences in collagen density as detected by dual-energy computed tomography (DECT) of wrist ligaments between patients with calcium pyrophosphate-dihydrate deposition disease (CPPD) and a control group in order to gain insight into changes of the extracellular matrix in response to crystal deposition.

**Materials and methods:**

This retrospective study included 28 patients (18 with CPPD, 10 controls) who underwent DECT of the wrist. Collagen density maps were reconstructed from the DECT datasets and used to measure densities in regions of interest (ROIs) placed in the scapholunate (SL) ligament (dorsal, palmar, proximal), lunotriquetral (LT) ligament, and extensor carpi radialis (ECR) tendon, (*n* = 260 measurements). The presence of calcifications on standard CT images in these regions was assessed by a blinded reader. Densities were compared with nonparametric tests, and linear regression analysis was performed to investigate the impact of age, sex, and CT- detected calcium deposition on collagen density.

**Results:**

Collagen density in the SL ligament was significantly higher in CPPD patients than in controls (overall mean: 265.4 ± 32.1 HU vs. 196.3 ± 33.8 HU; *p* < 0.001). In the ECR tendon, collagen densities did not differ significantly (*p* = 0.672): 161.3 ± 20.1 HU in CPPD vs. 163.6 ± 12.0 HU in controls. Regression analysis showed that diagnosis, but not age or calcification, had a significant impact on collagen density.

**Conclusion:**

Collagen density of the SL ligament is significantly higher in CPPD patients than in control patients. Further research is needed to understand these changes in the extracellular matrix of ligaments in CPPD.

## Introduction

Calcium pyrophosphate dihydrate crystal deposition disease (CPPD) has been described as the third most common inflammatory arthritis [1], affecting predominantly the elderly [2]. CPPD presents with a variety of clinical symptoms ranging from oligo- or asymptomatic calcium deposition to acute painful arthritis with findings of systemic inflammation [3]. Depositions are found predominantly in the cartilage, although involvement of ligamentous structures has long been established [4]. A known complication of CPPD of the wrist is rupture of the scapholunate (SL) ligament and development of a scapholunate advanced collapse (SLAC) pattern of degeneration [5, 6]. A recent investigation using low-dose CT identified the SL ligament as a site commonly affected by CPPD [7].

Presently, CPPD is frequently diagnosed on plain radiographs [8], although much recent research has focused on the role of ultrasound [9, 10]. Another promising imaging tool in the evaluation of crystal arthropathies is dual-energy computed tomography (DECT) [11]. While DECT was traditionally mainly used for imaging in gout [12], recent studies have critically evaluated its capacity to differentiate between different forms of crystal arthropathy [13] and demonstrated its ability to visualize bone marrow edema [14, 15]. Another pioneering application of the technique has been the visualization of collagen, first demonstrated by Johnson et al. [16]. Building on these results, a number of investigations have shown collagen mapping using DECT to be feasible in the imaging of the ligaments of the knee [17, 18] and the tendons of the hands and feet [19].

The aim of this investigation was—for the first time—to evaluate collagen density of selected ligaments in patients with known CPPD compared with patients with other types of arthritis of the hand in order to gain insight into interstitial changes in response to crystal deposition.

## Materials and methods

### Patients

We retrospectively included patients who underwent a single-source dual-energy computed tomography (SDECT) scan of the wrists for suspected crystal arthropathy in the Department of Radiology of our university hospital. Patients with known CPPD according to the McCarty [20] criteria were assigned to the CPPD group, other patients to the control group. Patient age, sex, and diagnosis were recorded in a pseudonymized manner. The local ethics review board waived approval of this study due to its retrospective nature. All patients gave written informed consent to the scientific use of imaging and clinical data prior to the CT examination, which is a common procedure at our institution.

### Imaging technique

All patients underwent a low-dose dual-energy CT scan of both wrists (one patient had only one wrist examined) in a 320-row CT scanner (Canon Aquilion One and Canon Aquilion One Vision, Canon Medical Systems, Otawara, Japan), using a standardized scan protocol. Tube settings were as follows: 80 kVp with 90 to 170 mA and 135 kVp with 15 to 30 mA. We used the volume mode with a z-axis coverage of 16 cm without table movement and with the fastest tube rotation time. To establish a conventional CT image visualization, virtual blended 120 kVp equivalent images were computed as well as collagen maps using a vendor software (Canon Medical Systems, Otawara, Japan) on the CT console and applying a dual-energy gradient of 1.1 for collagen on the three-material decomposition software. Readers were blinded to all patient data, which was ensured by pseudonymization with dedicated software (Horos v.2.2.0, The Horos Project).

### Measurements

**Fig. 1:**
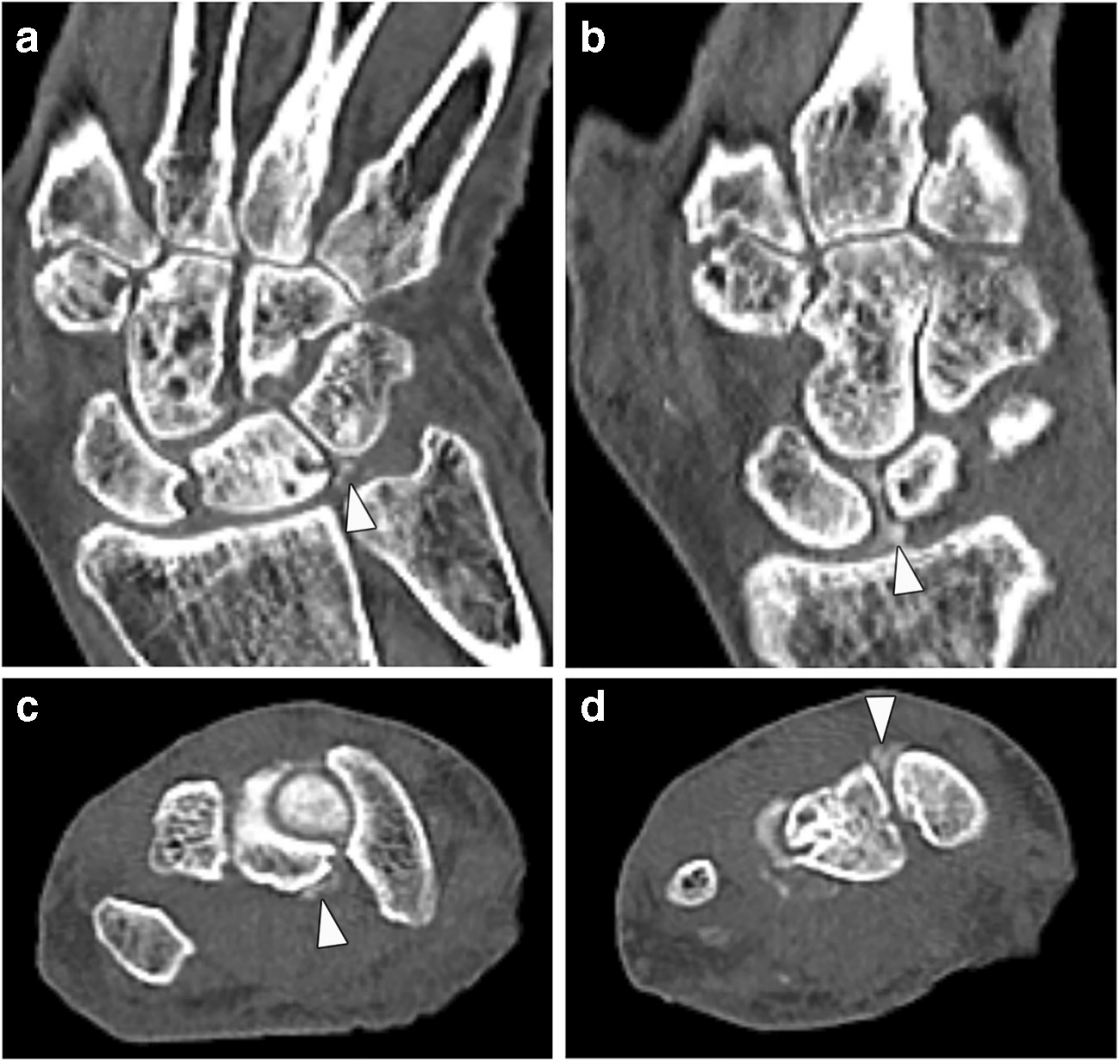
Imaging examples: ligamentous calcifications. Computed tomography of an 80-year-old female patient with CPPD; 80 keV bone kernel reconstructions. **a** Coronal reconstruction; arrowhead indicating calcification in proximal portion of the LT ligament. **b** Coronal reconstruction; arrowhead indicating calcification in proximal portion of the SL ligament. **c** Axial reconstruction; arrowhead indicating calcification in palmar portion of the SL ligament. **d** Axial reconstruction; arrowhead indicating calcification in dorsal portion of the SL ligament

**Fig. 2:**
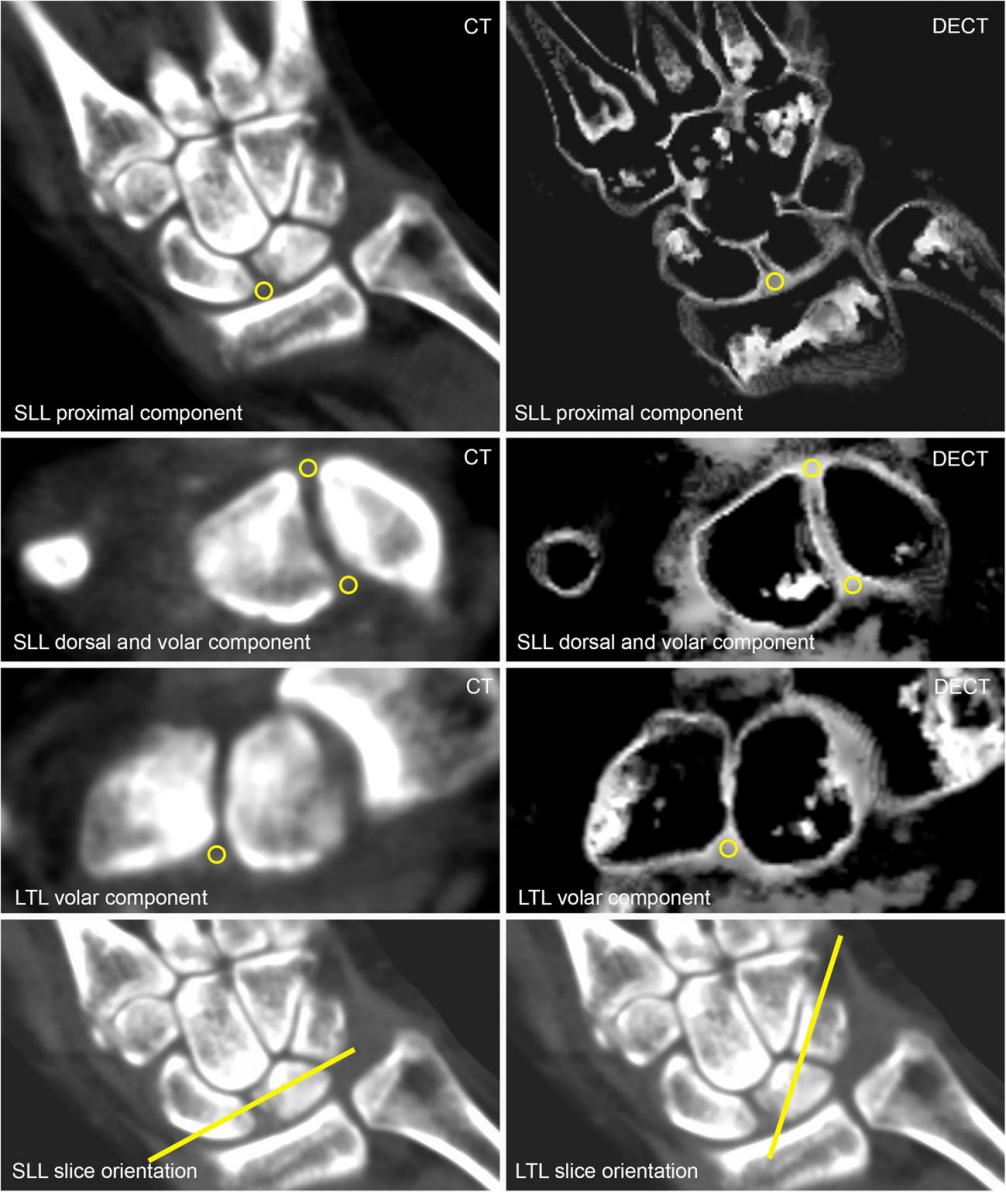
Measurement of collagen density. All images are from a 61-year-old female patient with gout. Left column: equivalent 120 kVp blended CT images. Right column: collagen maps. First row: example of ROI placement in proximal component of SL ligament. Second row: example of ROI placement in dorsal and volar component of SL ligament. Third row: example of ROI placement in volar component of LT ligament. Last row (both equivalent 120 kVp blended CT images): example of slice orientation for measurements

Ligamentous calcifications were scored dichotomously (present or absent) on the virtual blended images in the dorsal, palmar, and proximal portions of the scapholunate (SL) ligament, the palmar portion of the lunotriquetral (LT) ligament; they were defined as linear or flake-like hyperdensities within the ligament (imaging example given in Fig. 1). This evaluation was performed by a radiological resident (KZ) and additionally by a senior radiologist (TD), in order to calculate inter-reader agreement—both readers were blinded to all clinical data. Additionally, region of interest (ROI) measurements were performed on collagen maps by a specially trained research student (STR), who noted mean densities (HU) and standard deviations (SDs) at the ligamentous sites described above as well as the extensor carpi radialis (ECR) tendon, which was chosen as a control region as it has not been described as a common site of involvement in CPPC in systematic evaluations. A measurement was noted as missing when the anatomical region was affected by extensive calcifications (obscuring significant portions of the ligament) on standard CT images or not evaluable due to other patient-related factors (e.g., ankylosis). An example illustrating ROI placement is provided in Fig. 2.

### Statistical analysis

All statistical analyses were carried out with SPSS Version 25 (IBM Corporation, New York, USA). Differences in distribution of nominal variables were investigated using Fisher’s exact test; differences in continuous variables were compared using either Mann-Whitney *U* tests or *T* tests, depending on the results of previously administered Shapiro-Wilks tests. Furthermore, impact of age, sex, ligamentous calcification (if detected by both readers in one or more regions of the ligament), and diagnosis was investigated using a linear regression model. Agreement regarding presence of ligamentous calcifications was calculated using Cohen’s kappa. For all tests, a *p* < 0.05 was assumed to indicate statistical significance.

## Results

### Patients

Twenty-eight patients were included into this case-control study, 18 in the CPPD group (classified as “probable CPPD” according to the McCarty criteria [20]) and 10 in the control group (gout *n* = 4; healthy *n* = 3; rheumatoid arthritis *n* = 2; osteoarthritis *n* = 1). The CPPD group included 8 men (44.4%); the control group included 6 men (60.0%); the difference in distribution was not statistically significant (*p* = 0.695). Patients in the CPPD group were significantly (*p* = 0.002) older than patients in the control group: 69.6 (SD 6.9) years in the CPPD group vs. 56.8 (SD 12.4) years in the control group.

### Descriptive analysis

**Fig. 3:**
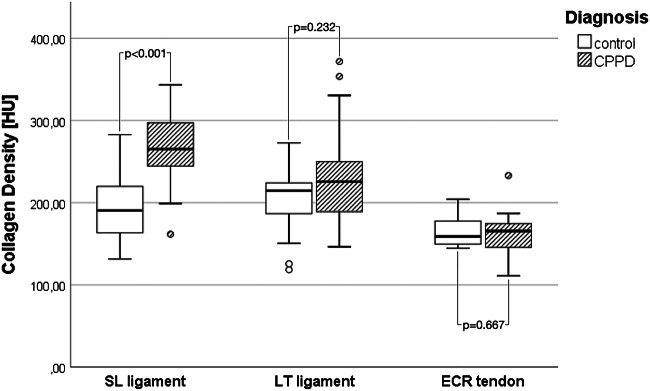
Boxplots of collagen densities. Left boxes, SL ligament. Middle boxes, LT ligament. Right boxes, ECR tendon. White boxes, controls. Hashed boxes, CPPD. *p* values derived from Mann-Whitney *U* test

**Fig. 4:**
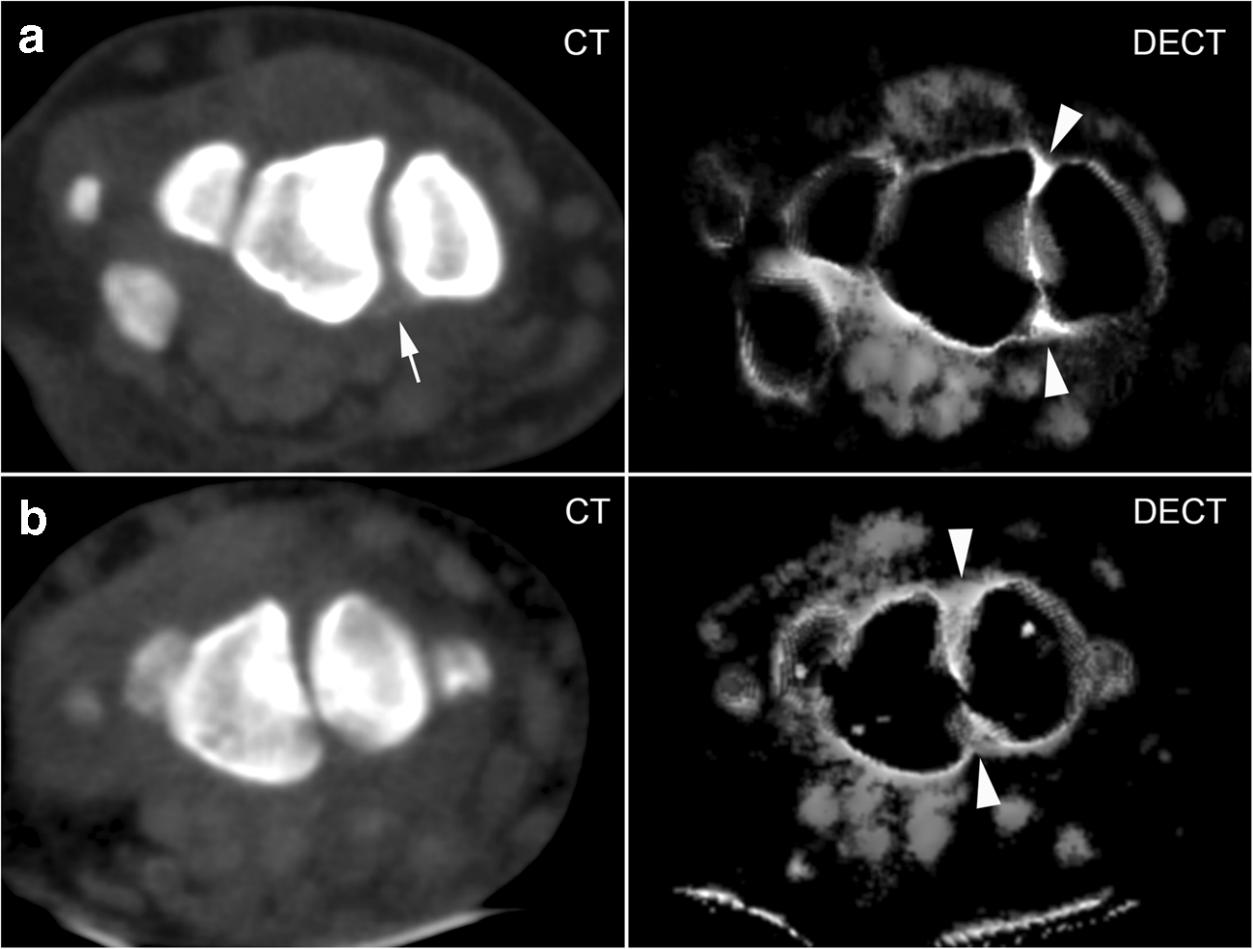
Imaging examples. Row A: 58-year-old male patient with CPPD: equivalent 120 kVp blended image (left) and DECT collagen map (right). Row B: 70-year-old male patient with late-onset rheumatoid arthritis: equivalent 120 kVp blended image (left) and DECT collagen map (right). White arrow indicates calcification; white arrow heads indicate areas with higher collagen density (in the dorsal and palmar aspects of the SL ligament) in the CPPD patient

A total of 260 individual collagen density measurements were recorded—another 20 measurements could not be included due to macroscopic calcification (*n* = 5), ankylosis (*n* = 3), and SL ligament rupture (*n* = 6), or because the region was not included in the scan (*n* = 6). Frequencies of depositions on virtual blended images in each location are compiled in Table 1—calcium depositions were significantly more prevalent in the CPPD group in all localizations except the right palmar aspect of the SL ligament. Mean collagen density of the ECR tendon did not differ between CPPD (161.3 HU; SD 20.1) and control patients (163.6 HU; SD 12.0) with *p* = 0.672. In all three compartments of the SL ligament, mean collagen density was significantly higher in the CPPD group than in the control group (see Table 1): mean density of the dorsal part was 264.2 (SD 55.0) HU in the CPPD group vs. 207.3 (SD 50.9) HU in the control group (*p* = 0.001), while it was 259.2 (SD 47.2) HU in the palmar portion in CPPD vs. 185.5 (31.6) HU in controls (*p* < 0.001) and 269.9 (SD 60.2) HU in the proximal portion in CPPD vs. 185.8 (SD 52.3) HU in controls (*p* < 0.001). A boxplot of mean collagen densities of the entire SL ligament (averaged from the measurements reported above) and the ECR tendon is presented in Fig. 3. Mean density of the LT ligament on collagen maps was 227.0 HU (SD 51.7) in CPPD patients vs. 206.6 HU (SD42.8) in control patients—the difference was not statistically significant (*p* = 0.223). An imaging example of collagen maps in two representative patients is shown in Fig. 4. Inter-reader agreement, as expressed by Cohen’s kappa, was strong with a mean kappa of 0.66 (range: 0.55–0.77; *p* < 0.001).

**Table 1:** Frequencies of calcium depositions and mean collagen densities. Frequencies (as detected on equivalent 120 kVp blended images) are given as percentages with absolute numbers in parentheses. Collagen densities are given as means and standard deviations. *p* values were derived from Fisher’s exact test (frequencies) and Mann-Whitney *U* tests (mean collagen densities). Significantly higher frequencies are marked with asterisks

Anatomic structure		Frequency of calcium deposition			Mean collagen density		
		CPPD (%, *n*)	Control (%, *n*)	*p*	CPPD (HU, SD)	Control (HU, SD)	*p*
SL ligament-dorsal	Right	56% (10/18)*	0% (0/10)	0.004	279.2 ± 48.6*	206.1 ± 53.0	0.003
	Left	50% (9/18)*	0% (01/10)	0.010	249.2 ± 58.4	208.6 ± 51.6	0.084
SL ligament-palmar	Right	44% (8/18)	10% (1/10)	0.098	268.3 ± 38.0*	187.4 ± 36.7	< 0.001
	Left	56% (10/18)*	0% (0/10)	0.004	244.7 ± 68.4*	183.3 ± 26.8	0.001
SL ligament-proximal	Right	39% (7/18)	10% (1/10)	0.194	258.8 ± 59.9*	192.2 ± 59.3	0.006
	Left	67% (12/18)*	0% (0/10)	0.001	281.8 ± 66.0*	178.7 ± 45.5	0.001
LT ligament	Right	61% (11/18)*	0% (0/10)	0.002	239.7 ± 55.1*	189.4 ± 46.3	0.016
	Left	72% (13/18)*	0% (0/10)	< 0.001	214.4 ± 46.1	225.6 ± 30.6	0.403

### Impact of age, sex, and calcification

The linear regression model for mean SL ligament collagen density (all regions, both sides) had an *R*^2^ of 0.49 (*p* < 0.001); the only significant coefficient was the diagnosis of CPPD with a beta of 0.50 (*p* = 0.001), while neither age (beta = 0.20; *p* = 0.112) nor calcium deposition in CT (beta = 0.010; *p* = 0.934) had a significant impact on collagen density. The model for mean LT ligament collagen density had an *R*^2^ of 0.06 (*p* = 0.508); none of the coefficients were significant: diagnosis (beta = 0.172; *p* = 0.389), age (beta = 0.150; *p* = 0.393), and calcium deposition in CT (beta = −0.105; *p* = 0.563) therefore had no impact on collagen density of the LT ligament. The model for the ECR tendon (both sides) yielded an *R*^2^ of 0.159 (*p* = 0.033)—diagnosis had a beta of − 0.295 (*p* = 0.068) while age had a beta of 0.504 (*p* = 0.004).

## Discussion

This study was conducted to investigate collagen density of carpal ligaments in patients with CPPD in comparison with a group of controls with other forms of arthritis using dual-energy CT. We found significantly higher amounts of collagen in the SL ligaments of patients suffering from CPPD.

These findings are somewhat surprising, as deposition of calcium and subsequent inflammation or resorption have so far been assumed to decrease extracellular matrix, thus destabilizing the ligament. One possible explanation may be that larger amounts of collagen indicate less organized tissue remodeling with fewer elastic fibers and therefore less dynamic stability. This may be understood as an expression of excessive or non-physiological stress. A larger volume of ligamentous structures has been described in (incidental) crowned dens syndrome, which is characterized by retro-odontoid thickening [21]. A possible alternative explanation could be that the increased density in collagen maps is attributable to a partial volume effect of microscopic calcium depositions. However, despite similar calcium deposition, the LT ligament showed no increase in collagen density which makes this explanation less likely. Furthermore, the applied three-material decomposition algorithm for the reconstruction of the collagen maps accounts for those effects. In summary, we believe the most likely explanation for our findings is a remodeling of the extracellular matrix in response to crystal deposition.

The importance of the SL ligament for the biomechanical stability of the proximal carpal row has long been established [22], and SL rupture with subsequent development of a SLAC wrist pattern of osteoarthritis is considered an imaging marker of CPPD [23]. Our findings suggest that extracellular changes in response to calcium deposition differ somewhat between the ligaments of the wrist. We therefore conclude that DECT may have clinical applications in earlier disease stages of CPPD, depicting tissue remodeling before SL rupture manifests—however, further research is necessary to better understand variations in collagen densities in ligamentous structures of the wrist. Thus far, depiction of traumatized ligaments by DECT has only been demonstrated in the anterior cruciate ligament [24] and collagen imaging has only been applied in visualization of postoperative knees [25]. Our control patients were statistically significantly younger than our CPPD patients (on average by 12.8 years). Using regression analysis, we investigated the impact of age on collagen content of ligamentous structures and we did not find a significant connection for ligaments, but an increase in collagen with age in tendons. The latter finding is in line with histopathological studies investigating the aging process of tendons [26]. We therefore conclude that, although the age difference somewhat limits the comparability of our patient group and our control group, our results seem valid.

There are some limitations to this investigation which need to be discussed. Firstly, the results presented here were obtained in a small group of patients, which was recruited retrospectively and also in most cases without direct crystal demonstration. Secondly, blinding to the diagnosis was not possible in all cases, as extensive ligamentous calcifications were apparent in collagen maps. Lastly, we used the ECR tendon as a reference structure under the assumption that calcific deposits in this area are rare, as they have not been systematically investigated—there is ample evidence in the literature, however, of tendon involvement in CPPD [27–29].

In conclusion, we have demonstrated that collagen mapping of ligamentous structures reveals significant differences between patients with CPPD and a control group in the SL ligament. In the future, DECT may facilitate the detection of microstructural changes in ligaments and tendons in other conditions (e.g., trauma, rheumatoid arthritis) as well.
